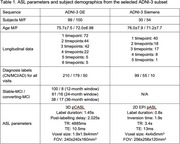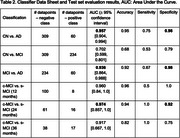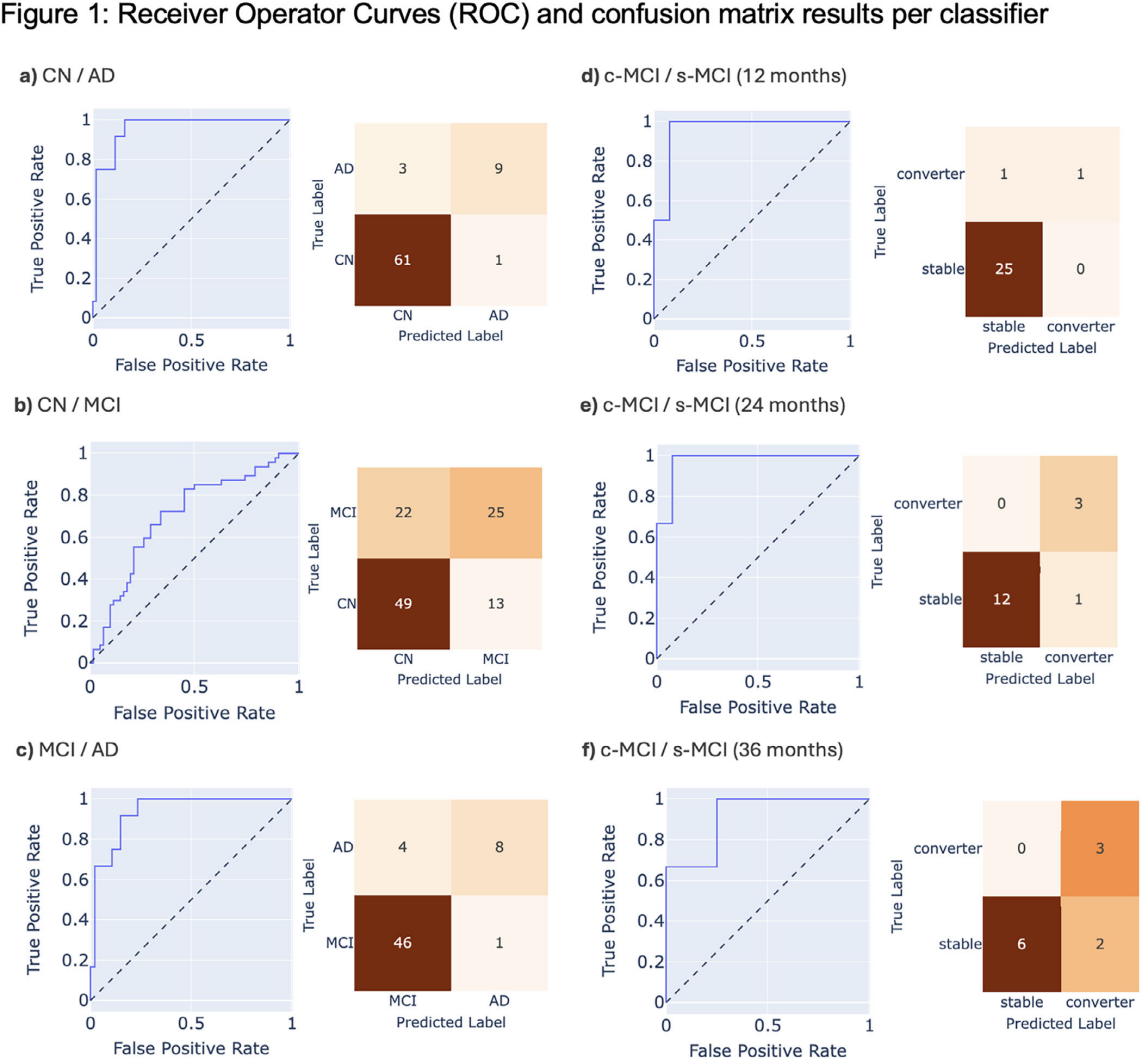# Arterial spin labelling MRI predicts diagnosis label and progression on ADNI‐3 data

**DOI:** 10.1002/alz70856_099000

**Published:** 2025-12-24

**Authors:** Armen Bodossian, Avital Dell'Ariccia, Logan Xin Zhang, Thomas Kirk, Martin Craig, Michael Chappell

**Affiliations:** ^1^ Quantified Imaging, London, London, United Kingdom; ^2^ University of Nottingham, Nottingham, Nottinghamshire, United Kingdom

## Abstract

**Background:**

Arterial spin labelling (ASL) MRI permits non‐invasive cerebral blood flow (CBF) measurement. Previous work has associated regional hypo‐perfusion with Alzheimer's disease (AD) progression prior to brain atrophy^1^. This study assesses the use of ASL for AD diagnosis and progression.

**Method:**

We selected a subset of Alzheimer's Disease Neuroimaging Initiative (ADNI‐3) subjects (Table 1), with ground‐truth diagnostic label (cognitively normal CN, mild cognitive impairment MCI, and AD). For MCI subjects with longitudinal data, we defined stable‐MCI (s‐MCI) as never converting to AD, and converting‐MCI (c‐MCI) as converting within 12, 24 or 36 months. ASL images were processed using the *qasl* pipeline (Quantified Imaging, London, UK), which follows the principles outlined by Alsop^2^ using a Bayesian algorithm^3^ to obtain calibrated CBF maps. T1‐weighted images were segmented using FreeSurfer to obtain ROI‐based CBF. To harmonise across acquisitions, CBF was normalised using the pallidum.

Six binary classifiers were trained using ROI‐based CBF, covariates (age, sex, education) and scanner type. Feature selection was driven by ANOVA between diagnostic groups. A support vector machine (SVM) was used to predict diagnosis.

Data was split into a 80:20 train‐test set, stratified by scanner and diagnosis. Hyperparameters were tuned with n‐1 folds cross‐validation. The model with the highest average F1‐score was trained and the probability threshold was optimised using Youden's index. Performance was evaluated on the test set.

**Result:**

Results are summarised in Table 2, with ROC curves and confusion matrices in Figure 1. Classifiers distinguished between AD vs. CN or MCI with AUCs of 0.957 [95% CI: 0.904, 0.994] and 0.936 [0.864, 0.988], respectively, both achieving specificity of 0.98. CN vs. MCI classification was less performant (AUC 0.702 [0.599, 0.801]). For c‐MCI vs. s‐MCI, AUC exceeded 0.9 for all timeframes.

**Conclusion:**

We demonstrated that ASL measures could predict clinical diagnosis and disease progression, outperforming both PET^4^ and previous ASL^5^ classifiers reported in literature in similar analyses.

**References**

1. Johnson et al., *Radiology*. 2005;234(3):851‐859. doi:10.1148/radiol.2343040197

2. Alsop et al., *Magn Reson Med*. 2015;73(1):102‐116. doi:10.1002/mrm.25197

3. Chappell et al. Imaging Neuroscience. 2023;1:1‐16. doi:10.1162/imag_a_00041

4. Bicacro et al.,19th IEEE International Conference on Image Processing; 2012;1237‐1240. doi:10.1109/ICIP.2012.6467090

5. Díaz et al., *CIARP*, 2014; 2014:714‐721. doi:10.1007/978‐3‐319‐12568‐8_87